# Short tandem repeat genotyping of *Pichia kudriavzevii* isolates reveals long-standing clinical clade with high transmission potential

**DOI:** 10.1093/mmy/myag082

**Published:** 2026-08-06

**Authors:** Bram Spruijtenburg, Kusum Jain, Merlijn H I van Haren, Jacques F Meis, Theun de Groot, Eelco F J Meijer, Anuradha Chowdhary

**Affiliations:** Department of Medical Microbiology, Radboudumc, 6525 GA, Nijmegen, The Netherlands; Radboudumc-CWZ Center of Expertise for Mycology, 6532 SZ, Nijmegen, The Netherlands; Department of Medical Microbiology and Immunology, Canisius-Wilhelmina Hospital (CWZ)/Dicoon, 6532 SZ, Nijmegen, The Netherlands; Medical Mycology Unit, Department of Microbiology, Vallabhbhai Patel Chest Institute, University of Delhi, 110007, Delhi, India; Department of Medical Microbiology, Radboudumc, 6525 GA, Nijmegen, The Netherlands; Radboudumc-CWZ Center of Expertise for Mycology, 6532 SZ, Nijmegen, The Netherlands; Department of Medical Microbiology, Radboudumc, 6525 GA, Nijmegen, The Netherlands; Radboudumc-CWZ Center of Expertise for Mycology, 6532 SZ, Nijmegen, The Netherlands; Institute of Translational Research, Cologne Excellence Cluster on Cellular Stress Responses in Aging-Associated Diseases (CECAD) and Excellence Center for Medical Mycology, University of Cologne, 50923, Cologne, Germany; Radboudumc-CWZ Center of Expertise for Mycology, 6532 SZ, Nijmegen, The Netherlands; Department of Medical Microbiology and Immunology, Canisius-Wilhelmina Hospital (CWZ)/Dicoon, 6532 SZ, Nijmegen, The Netherlands; Department of Medical Microbiology, Radboudumc, 6525 GA, Nijmegen, The Netherlands; Radboudumc-CWZ Center of Expertise for Mycology, 6532 SZ, Nijmegen, The Netherlands; Department of Medical Microbiology and Immunology, Canisius-Wilhelmina Hospital (CWZ)/Dicoon, 6532 SZ, Nijmegen, The Netherlands; Medical Mycology Unit, Department of Microbiology, Vallabhbhai Patel Chest Institute, University of Delhi, 110007, Delhi, India; National Reference Laboratory for Antimicrobial Resistance in Fungal Pathogens, Vallabhbhai Patel Chest Institute, University of Delhi, 110007, Delhi, India

**Keywords:** *Pichia*, *Candida*, antifungal resistance, outbreaks, nosocomial transmission, genotyping

## Abstract

*Pichia kudriavzevii* (previously known as *Candida krusei*) is among the top five causative agents of candidemia, mainly affecting neonates. The yeast is intrinsically resistant to fluconazole, and nosocomial transmission has been reported. Recently, whole genome sequencing (WGS) analysis on 24 out of 165 clinical *P. kudriavzevii* isolates from Delhi, India showed two clusters, indicating nosocomial transmission. To further increase our insight into the transmission of this fungus in healthcare settings, we performed short tandem repeat (STR) genotyping of 106 isolates from Delhi, India, and compared outcomes to STR genotypes and WGS data of isolates originating from various countries. A total of 52 unique genotypes were identified, in addition to five clusters comprising two to 22 isolates. Four of these clusters were restricted to a single hospital, while the largest cluster included six hospitals. Interestingly, when compared to a global collection, Iranian clinical isolates were identical to the multicenter cluster and also related to isolates from the Netherlands and Sri Lanka, the latter isolate being collected in 1935. WGS analysis on a selection of isolates confirmed the clusters identified by STR analysis, underscoring the high discriminatory power of the STR genotyping scheme. In short, we report several events of single-center nosocomial transmission of *P. kudriavzevii* in India. One multicenter cluster was found, which was closely related to isolates originating from diverse countries, suggesting a fitness advantage or elevated transmission potential for this lineage.

## Introduction

Candidemia is among the most common invasive fungal infections with an estimated annual incidence of 1.5 million cases.^1^ Candidemia mainly occurs in immunocompromised patients with risk factors such as admission to the intensive care unit (ICU), undergoing chemotherapy, or having immunodeficiency disorders.^2^ Given that this particular patient population expanded considerably in the last three decades, the prevalence of candidemia episodes is increasing globally, placing a significant burden on healthcare.^2^ Until recently, *Candida albicans* was the most common etiological agent, with *C. tropicalis, Nakaseomyces glabratus* (synonym *C. glabrata*), and *C. parapsilosis* observed as well.^3^ However, a worrying shift toward non-*C. albicans* species has been observed lately. These species are more often resistant to antifungal agents, having a greater outbreak-causing potential and pose diagnostic challenges especially infections caused by rare yeast species.^4^

*Pichia kudravzevii* (previously known as *C. krusei*) ranks as the fifth most common causative agent of candidemia, and is known for its intrinsic antifungal resistance to fluconazole.^5,6^ Even though echinocandins are the drugs of choice to treat candidemia, fluconazole is often the only affordable antifungal in developing countries.^2^ Therefore, clinical failure of *P. kudriavzevii* candidemia episodes is commonly observed.^7^ Resistance to other agents such as amphotericin B and echinocandins is occasionally reported.^7^

Neonates are often affected by *P. kudriavezvii*, with low birth weight and premature delivery as main risk factors for candidemia.^8^ In addition, outbreaks by *P. kudriavzevii* mainly occur in neonatal ICUs and wards with suboptimal infection prevention measures.^8–10^ To investigate nosocomial transmission, high-resolution genotyping methods like whole genome sequencing (WGS) or short tandem repeat (STR) analysis are required to uncover genetic relatedness between isolates.^11^ Alternative methods include multilocus sequence typing and amplified fragment length polymorphism.^12^ While the first is known for its high reproducibility and the latter for its short turnaround time, discriminatory power of both is often insufficient to accurately label isolates as clonal. To date, few studies, on genetic diversity of *P. kudriavzevii*, have been reported, sometimes showing large clusters while others found limited relatedness between isolates.^10^ Recently, a retrospective study investigated the antifungal susceptibility of clinical *P. kudriavzevii* isolates and performed WGS analysis on a selection of 28 out of 165 isolates.^13^ While most tested drugs were effective *in vitro*, 13 non-wild-type isolates for itraconazole were found. Moreover, WGS identified two clonal lineages, demonstrating nosocomial transmission. In the current study, we therefore investigated a large dataset of isolates of *P. kudriavzevii* with STR genotyping and compared the outcome with antifungal resistance and publicly available WGS data.

## Material and methods

A total of 106 *P. kudriavzevii* isolates, collected between 2015 and 2019 in Delhi, India, were studied.^13^ Of these, 59/106 isolates were from neonates, followed by 30 isolates from pediatric patients and 16 from adults, most with fungemia. Isolates were stored according to standard procedures at −80°C.

### STR genotyping

For STR genotyping, isolates were taken from storage and grown on Sabouraud dextrose agar plates (Oxoid, Basingstoke, UK) at 35°C for 48 h. At the Radboudumc-CWZ Center of Expertise for Mycology, Nijmegen, The Netherlands, DNA was extracted from a single colony with the MagNA Pure 96 (Roche Diagnostics GmbH, Mannheim, Germany) according to manufacturer’s instructions as described in detail earlier.^14^ Next, six microsatellite alleles were amplified in two multiplex PCRs.^11^ Amplicons were diluted 1000 times in water and analyzed on a 3500 XL genetic analyzer (Applied Biosystems, Foster City, CA, USA). Copy numbers were determined with Genemapper 5 software (Applied Biosystems) and visualized using BioNumerics v7.6.1 (Applied Maths NV, Sint-Martens-Latem, Belgium) as described before.^15^ The current isolates were compared to previously genotyped isolates.^11,15,16,17^ The discriminatory power of each STR marker was calculated using the Simpson index of diversity (*D*), with a value of 1.0 indicating a unique allele for each isolate and a value of 0 indicating a similar allele across all isolates.^15^

### WGS single nucleotide polymorphism analysis

From the National Center for Biotechnology Information (NCBI) Sequence Read Archive (SRA), raw read data were extracted, and isolates were aligned against the *P. kudriavzevii* CBS 573 reference genome (GCA_003054445.1), followed by a single nucleotide polymorphism (SNP) analysis as previously described (Supplementary Table 1).^18^ In total, raw reads of 20 isolates were retrieved from NCBI under SRA accession numbers SRR28254453, SRR28254454, SRR28254455, SRR28254457, SRR28254458, SRR28254463, SRR28254464, SRR28254465, SRR28254466, SRR28254467, SRR28254468, SRR28254477, SRR28254478, SRR34361608, SRR34361609, SRR34361612, SRR34361613, SRR34361614, SRR34361615, and SRR34361616. In short, read mapping was conducted with BWA v0.7.19, and in the resulting BAM files, duplicates and unpaired reads were removed. SNPs were called using Freebayes v1.3.9, followed by extensive filtering. The inferred phylogeny was visualized using MEGA11 v11.0.10 and iTOL v6. Earlier, this SNP calling pipeline was validated with a benchmark dataset.^18^

## Results

### Genetic relatedness

**Figure 1.**
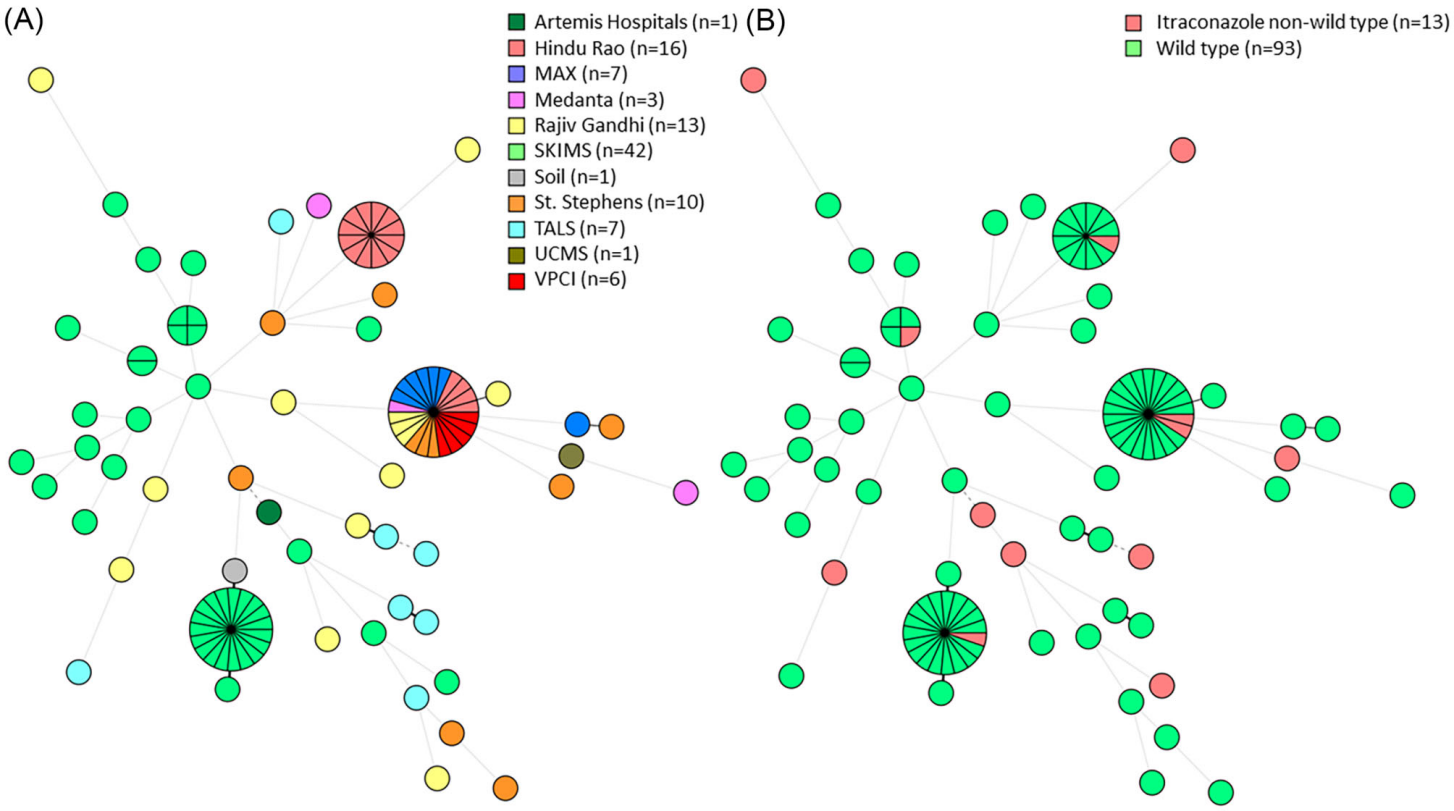
Minimum-spanning tree of 106 *P. kudriavzevii* isolates, colored according to hospital (A) or antifungal susceptibility (B). Branch lengths indicate the similarity between isolates with thick solid lines (variation in one allele), thin solid lines (variation in two alleles), thin dashed lines (variation in three alleles), and thin dotted lines (variation in four or more alleles).

From 2015 to 2019, 105 clinical *P. kudriavzevii* isolates and one soil sample were collected in Delhi, India.^13^ While clinical data and antifungal susceptibility testing (AFST) were reported earlier, we investigated the phylogenetic relatedness of these *P. kudriavzevii* isolates with STR genotyping. This resulted in 52 unique genotypes with five clusters comprising 2 to 22 isolates (Fig. 1A). Isolates from four smaller clusters were restricted to a single hospital and consisted of 2, 4, 12, and 19 isolates, respectively. The largest cluster included 22 isolates from six different hospitals. For the two largest clusters of 19 and 22 isolates, closely related genotypes were found that differed only one or two alleles. Interestingly, in the second largest cluster with 19 isolates, one of these closely related genotypes was collected from a soil sample, while 16 isolates were of clinical origin. Moreover, isolates from the largest cluster spanned a period of 4 years, while the other clusters originated in the same or 2 consecutive years. Besides the mentioned clusters, the genetic diversity was high with most genotypes differing by four or more microsatellite alleles from each other. The discriminatory power of each marker (*D*-value) was provided in Supplementary Table 2.

Next, we compared the genotyping results with earlier reported AFST data.^13^ In that report, 13 out of these 106 isolates were classified as non-wild type for itraconazole with two to four dilutions above the cutoff value (Supplementary Fig. 1). These itraconazole non-wild-type isolates did not cluster but rather demonstrated a wide distribution over 11 different genotypes (Fig. 1B). Furthermore, five non-wild-type isolates had an identical STR genotype as wild-type isolates.

### Global genetic diversity of *P. kudriavzevii*

**Figure 2.**
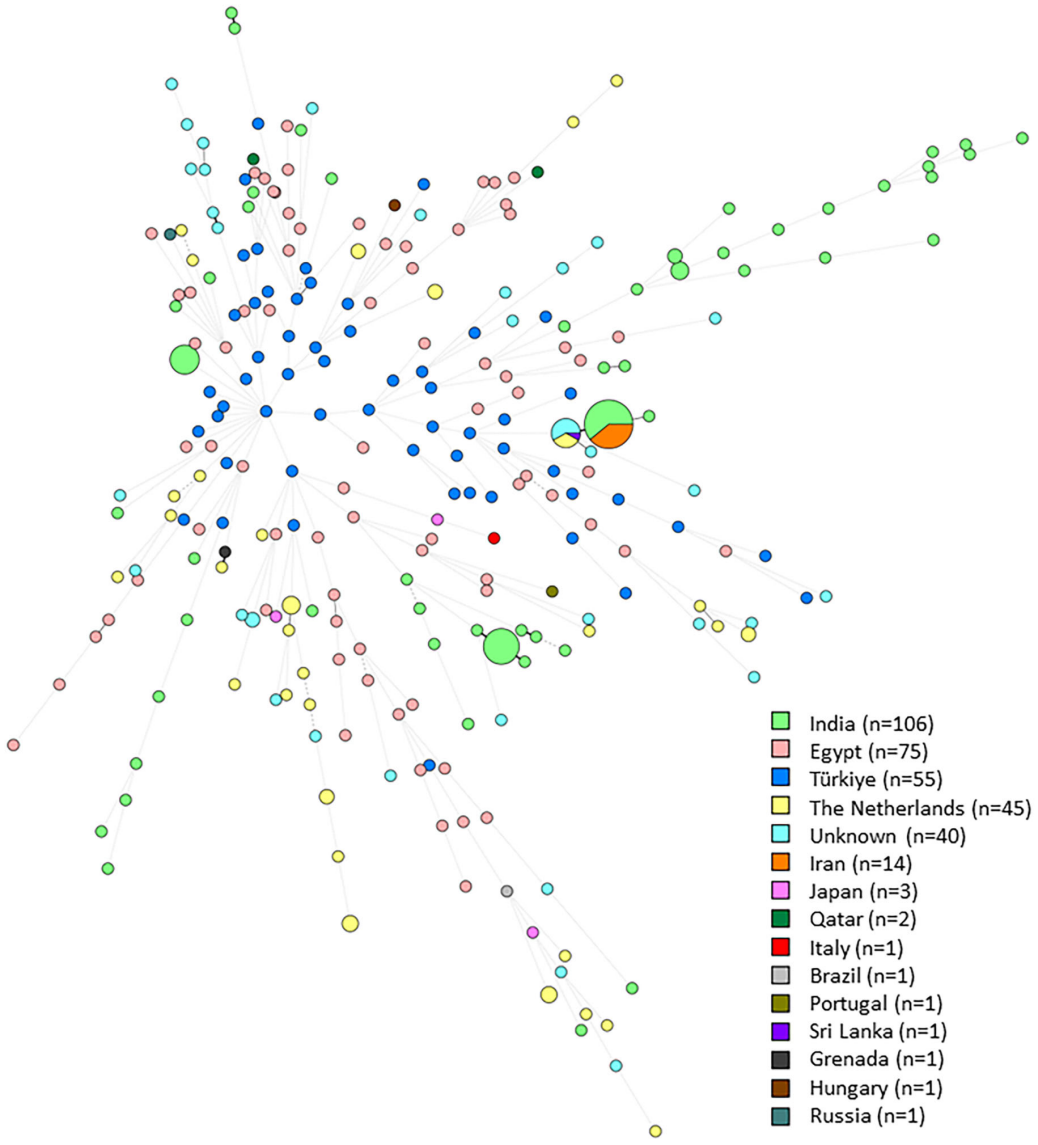
Minimum-spanning tree of 347 *P. kudriavzevii* isolates genotyped in the current and previous studies, with isolates colored after the country of origin. Branch lengths indicate the similarity between isolates with thick solid lines (variation in one allele), thin solid lines (variation in two alleles), thin dashed lines (variation in three alleles), and thin dotted lines (variation in four or more alleles).

The 106 study isolates were compared to a global collection of 241 previously genotyped *P. kudriavzevii* isolates, mainly from Egypt, Türkiye, and the Netherlands showing a high genetic diversity (Fig. 2). Nearly all genotypes found in the current study differed in three STR alleles or more from previously published genotypes. In contrast, the multicenter cluster of 22 isolates from the current study was identical to 14 clinical isolates from Iran and differed only one allele to a cluster comprised of 12 isolates. This cluster consisted of seven isolates of unknown origin, four clinical isolates from the Netherlands, and a clinical isolate from Sri Lanka, isolated in 1935 and deposited in the Centraalbureau voor Schimmelcultures (CBS) collection as CBS 573.

**Figure 3.**
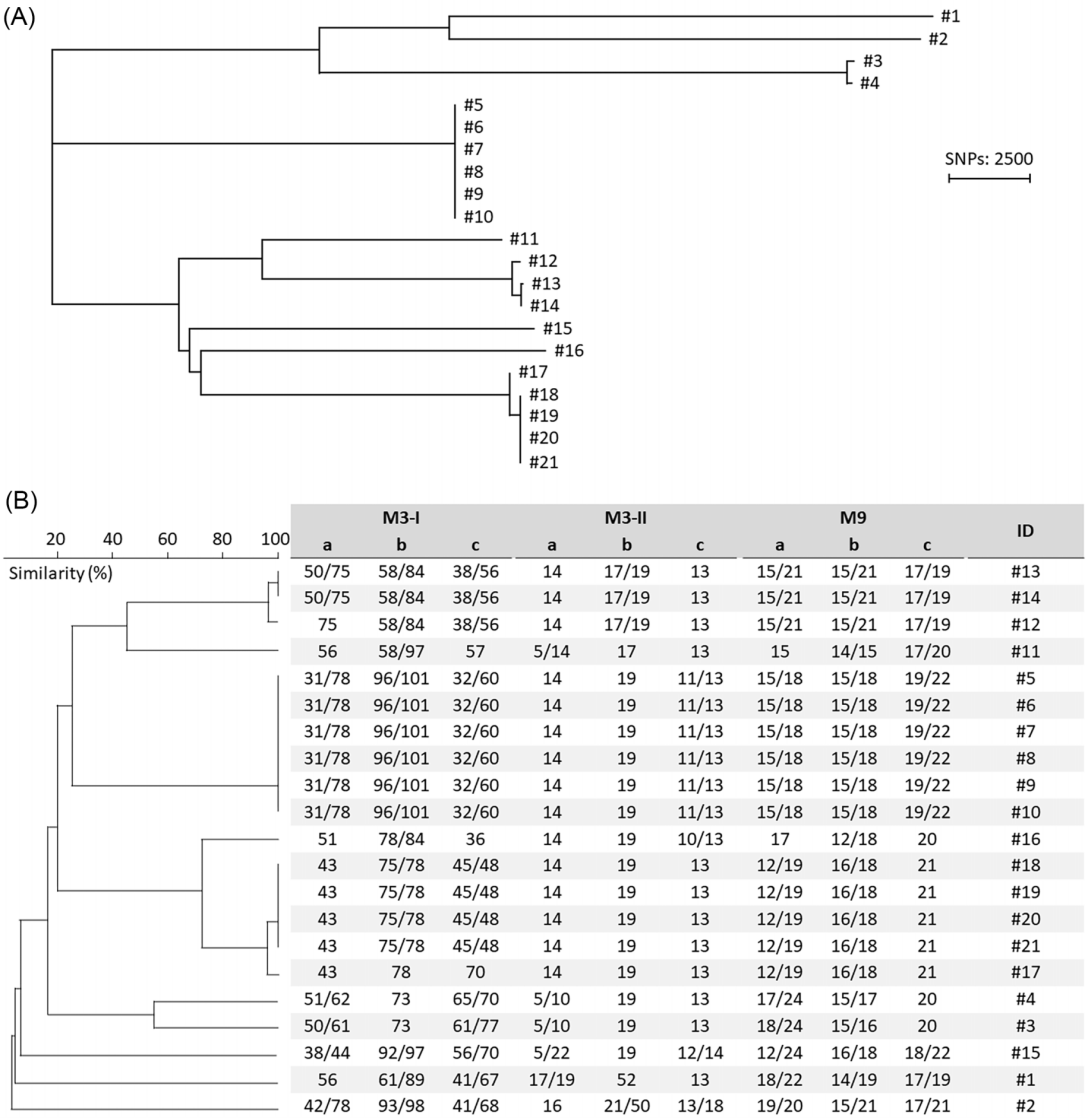
Genetic relatedness of 21 *P. kudriavzevii* isolates analyzed with whole genome sequencing (WGS) single nucleotide polymorphism (SNP) analysis (A) and short tandem repeat (STR) genotyping (B). Isolates are renamed for readability, #1: 10-11-09-10, #2: 10-04-13-30, #3: 1483/P/18, #4: 1461/P/18, #5: 1734/P/18, #6: 1596/P/18, #7: 577/P/19, #8: 601/P/19, #9: 581/P/19, #10: 723/P/17, #11: 123/P/19, #12: 7B(E), #13: 1381/P/18, #14: 1390/P/18, #15: 494/P/18, #16: 02/P/16, #17: CBS 573, #18: 1313/P/15, #19: 03/P/16, #20: 506/P/15, #21: 06/P/16.

To confirm the inferred phylogenetic relatedness, WGS SNP analysis was conducted on 21 previously sequenced isolates. Of these, 18 originated from one center in Delhi, India, and belonged to a total of 106 isolates, analyzed by STR in this study.^11^ The remaining three isolates originated from other investigations, mainly from India.^15^ Next, SNP outcome was compared to STR genotyping results. SNP differences between isolates ranged from 2 to 41 938 SNPs, confirming the high genetic diversity (Fig. 3A). Three groups of closely related isolates were found and differed 61 SNPs at most. Isolates 3 and 4 differed 512 SNPs, isolates 5–10 differed 48 SNPs at most, isolates 13 and 14 differed 216 SNPs, and isolates 18–21 differed 61 SNPs at most. With STR genotyping, three clusters of identical genotypes were identified, which coincided with the three groups found with WGS (Fig. 3B). Isolates that were separated by one or two STR alleles showed a difference of 511 SNPs at most.

## Discussion

This study investigated a large set of *P. kudriavzevii* isolates, mostly of neonatal origin. STR genotyping showed a high genetic diversity in addition to several large clusters, suggesting silent nosocomial transmissions. Comparison with a global collection demonstrated a diverse population structure but also revealed two closely related genotypes, comprising a total of 48 isolates from geographically diverse countries. Comparison with WGS SNP analysis showed a high discriminatory power of the STR genotyping scheme, with a difference of only 61 SNPs for isolates with identical genotypes.

With STR genotyping, 52 different genotypes were found among 106 *P. kudriavzevii* isolates, with 73% differing at least three or more STR markers from other genotypes. Earlier, this scheme was used to genotype isolates from various countries, including Türkiye and Egypt.^15,16^ Also in these studies, a diverse population structure was found, without the presence of large clusters, while large clusters have been found for other yeasts like *C. auris* and *C. parapsilosis*.^19,20^ This suggests that most *P. kudriavzevii* infections are acquired independently, while clonal transmission seems to play a minor role. Sources are probably local environmental niches, like the environmental isolate that was closely related to clinical isolates.^21^ Remarkably, multiple clusters were found in this study, in contrast to earlier investigations.^15,16^ Most of these clusters were restricted to single centers collected within the same or two consecutive years. Given that *P. kudriavzevii* is able to adhere and subsequently form biofilms on medical devices, hands of healthcare workers or medical equipment are implicated as the source of outbreaks.^9^ Similar to other outbreaks with rare yeasts like *Wickerhamomyces anomalus, C. vulturna*, and *Tardiomyces* (*Candida*) *blankii*, mainly neonates or pediatric patients are affected and often take place in low- and middle-income countries.^22–24^ The source generally remains elusive, and treatment is complicated due to intrinsic antifungal resistance, which applies to *P. kudriavzevii* as well.^5^ Fortunately, enhanced infection prevention measures and change of antifungal regimens are usually sufficient to curb these outbreaks.^25^

Notably, the largest cluster persisted during a 4-year period and affected six hospitals. Since *P. kudriavzevii* is extensively used in the food industry to facilitate fermentation,^26^ contaminated food distributed to multiple centers may be considered. However, Iranian samples displayed an identical genotype and were closely related to Dutch and Sri Lankan isolates, the latter collected nearly a century ago. This suggests that these isolates comprise a dominant lineage that is able to thrive within the human population due to a high transmission potential, which could be facilitated by strong adherence to inanimate surfaces.^27^ Follow-up studies might elucidate genomic factors underlying the elevated transmission potential of this multi-country lineage.

To validate the inferred genetic relatedness, WGS SNP data of a selection of isolates were compared to STR results. Isolates with identical STR genotypes only differed up to 61 SNPs at most, making both methods correlate well. Previous comparisons of WGS and STR genotyping noted similar findings, highlighting the high-resolution typing of STR assays in medical mycology.^11,28,29^

The main limitation of this study was its retrospective nature, which refrained us from collecting more environmental samples to trace the source of the clusters. Moreover, enhanced infection prevention and control measures could not be implemented to subsequently evaluate the efficacy. Next to a nosocomial source, environmental sampling could alternatively demonstrate that the source resides in a non-healthcare environment. In addition, medical history could not be retrieved, hindering our understanding of the spread and dynamics of the observed nosocomial transmission and correlating this information with genotyping results. Follow-up studies are needed to assess if the transmission was curbed or continues to date.

To conclude, we report a high genetic diversity of *P. kudriavzevii* in addition to several events of potential nosocomial transmission, often confined to a single hospital. However, one lineage of highly related genotypes was found in multiple hospitals and clinical Iranian, Dutch, and Sri Lankan isolates suggesting an increased transmission potential or fitness advantage in the human population.

## Supplementary Material

myag082_Supplemental_Files

## Data Availability

All data generated during this study is accessible via the main text or supplementary files.
