# Supplementary material for: Short tandem repeat genotyping of Pichia kudriavzevii isolates reveals long-standing clinical clade with high transmission potential
Source: Med Mycol. 2026 Aug 6;64(8):myag082. doi: 10.1093/mmy/myag082 (PMC13482982; doi:10.1093/mmy/myag082)
Supplement: Supplementary file 1 [file myag082_supplemental_files.zip › mm-2026-0222-File006.docx]

**Supplementary materials**

**Table S1: Overview of 21 *Pichia kudriavzevii* isolates extracted from the National Center for Biotechnology Information (NCBI) Sequence Read Archive (SRA).**

| **ID** | **Biosample** | **SRA** |
| --- | --- | --- |
| 10-04-13-30 | SAMN49785208 | SRR34361616 |
| 10-11-09-10 | SAMN49785209 | SRR34361615 |
| 506/P/15 | SAMN49785211 | SRR34361613 |
| 1313/P/15 | SAMN40266133 | SRR28254478 |
| 02/P/16 | SAMN40266136 | SRR28254457 |
| 03/P/16 | SAMN40266135 | SRR28254466 |
| 06/P/16 | SAMN40266134 | SRR28254477 |
| 494/P/18 | SAMN49785210 | SRR34361614 |
| 1381/P/18 | SAMN49785216 | SRR34361608 |
| 1390/P/18 | SAMN40266140 | SRR28254453 |
| 1461/P/18 | SAMN49785215 | SRR34361609 |
| 1483/P/18 | SAMN49785212 | SRR34361612 |
| 1596/P/18 | SAMN40266160 | SRR28254458 |
| 1734/P/18 | SAMN40266155 | SRR28254463 |
| 123/P/19 | SAMN40266138 | SRR28254455 |
| 577/P/19 | SAMN40266153 | SRR28254465 |
| 581/P/19 | SAMN40266151 | SRR28254468 |
| 601/P/19 | SAMN40266152 | SRR28254467 |
| 723/P/19 | SAMN40266154 | SRR28254464 |
| 7B(E) | SAMN40266139 | SRR28254454 |

**Table S2: Overview of discriminatory power (D-value) of each marker.**

| **PCR panel and marker** | ***D* value** |
| --- | --- |
| M3-1 | |
| M3-1a | 0.87 |
| M3-1b | 0.84 |
| M3-1c | 0.90 |
| M3-2 | |
| M3-2a | 0.82 |
| M3-2b | 0.86 |
| M3-2c | 0.88 |
| M9 | |
| M9a | 0.97 |
| M9b | 0.91 |
| M9c | 0.94 |
